# Cryopolymerization‐enabled self‐wrinkled polyaniline‐based hydrogels for highly stretchable all‐in‐one supercapacitors

**DOI:** 10.1002/EXP.20220006

**Published:** 2022-07-04

**Authors:** Hui Song, Yufeng Wang, Qingyang Fei, Dai Hai Nguyen, Chao Zhang, Tianxi Liu

**Affiliations:** ^1^ State Key Laboratory for Modification of Chemical Fibers and Polymer Materials College of Materials Science and Engineering Donghua University Shanghai China; ^2^ Key Laboratory of Synthetic and Biological Colloids Ministry of Education School of Chemical and Material Engineering International Joint Research Laboratory for Nano Energy Composites Jiangnan University Wuxi China; ^3^ Institute of Applied Materials Science Vietnam Academy of Science and Technology Ho Chi Minh City Vietnam

**Keywords:** all‐in‐one supercapacitors, cryopolymerization, polyaniline‐based hydrogels, stretchability, wrinkling surface

## Abstract

Conductive polymer hydrogels are attractive due to their combination of high theoretical capacitance, intrinsic electrical conductivity, fast ion transport, and high flexibility for supercapacitor electrodes. However, it is challenging to integrate conductive polymer hydrogels into an all‐in‐one supercapacitor (A‐SC) simultaneously with large stretchability and superior energy density. Here, a self‐wrinkled polyaniline (PANI)‐based composite hydrogel (SPCH) with an electrolytic hydrogel and a PANI composite hydrogel as the core and sheath, respectively, was prepared through a stretching/cryopolymerization/releasing strategy. The self‐wrinkled PANI‐based hydrogel exhibited large stretchability (∼970%) and high fatigue resistance (∼100% retention of tensile strength after 1200 cycles at a 200% strain) ascribing to the formation of the self‐wrinkled surfaces and the intrinsic stretchability of hydrogels. Upon cutting off the edge connections, the SPCH could directly work as an intrinsically stretchable A‐SC maintaining high energy density (70 µW h cm^−2^) and stable electrochemical outputs under a stretchability of 500% strain and a full‐scale bending of 180°. After 1000 cycles of 100% strain stretching and releasing processes, the A‐SC device could deliver highly stable outputs with high capacitance retention of 92%. This study might provide a straightforward method for fabricating self‐wrinkled conductive polymer‐based hydrogels for A‐SCs with highly deformation‐tolerant energy storage.

## Graphical abstract

It is challenging to develop new preparation methods of conductive polymer‐based composite hydrogels for constructing all‐in‐one supercapacitors (A‐SCs) with excellent deformation‐tolerant performance. Self‐wrinkled polyaniline‐based composite hydrogels (SPCHs) with large stretchability and excellent fatigue resistance are fabricated by a stretching/cryopolymerization/releasing strategy. The as‐obtained SPCH can work as an intrinsically stretchable A‐SC maintaining high energy density and stable electrochemical properties under deformations.

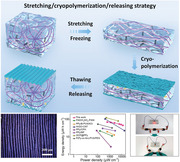

## 1 INTRODUCTION

Stretchable electronics with portable, lightweight, and wearable features have been extensively researched in the fields of human–machine interactions, health monitoring, and soft robotics.^[^ ^1^, ^2^, ^3^ ^]^ The preparation of deformation‐tolerant and high‐performance energy storage devices is significant for the power supply of these stretchable electronics.^[^ ^4^, ^5^ ^]^ Supercapacitors (SCs) feature the advantages of high power density, fast charging/discharging rate, and excellent cycle stability, and are promising energy storage devices in stretchable electronics.^[^ ^6^, ^7^ ^]^ However, traditional SCs based on electrode/electrolyte/electrode sandwiched architectures usually exhibit unsatisfactory stretchability and cannot meet the requirements of wearable and stretchable devices.^[^ ^8^ ^]^ As flexible energy storage devices and circuit elements, stretchable SCs that can be bent and stretched with high retention electrochemical performance have been widely investigated in recent decades.^[^ ^9^ ^]^ To date, two main methods have been proposed to construct stretchable SCs, one of which is to design strain‐accommodating geometries, including kirigami, wavy, and spring‐like structures for electrodes and devices to achieve stretchability.^[^ ^10^ ^]^ However, this fabrication method is complex and expensive, and the resultant devices usually have a confined reversible deformation range. The other is to deposit electrode materials onto stretchable substrates such as polydimethylsiloxane and polyurethane films to realize the extensibility of SCs.^[^ ^11^, ^12^ ^]^ However, stretchable substrates often occupy significant amounts of weight and volume, significantly reducing the energy density of devices.^[^ ^13^ ^]^ Moreover, stretchable SCs fabricated by the above methods demand the use of current collectors, electrodes, electrolytes, and separators to form a multilayer laminated configuration, which is not conducive to the rapid transmission of ions and electrons at the electrode/electrolyte interface, thereby inevitably increasing the interface contact resistances during deformation and reducing the Coulombic efficiency and cycle life of devices.^[^ ^14^ ^]^ In addition, the complex stretching, bending, and twisting deformations during practical applications of stretchable SCs would inevitably result in large displacements and delamination of the laminate, which severely attenuate device performance.^[^ ^15^ ^]^ Therefore, reasonable designs of molecular structures and cross‐linked networks of electrode and electrolyte layers to form strong interface interactions are of great value for the fabrication of stretchable SCs with high stretchability and excellent electrochemical performance.

All‐in‐one SCs (A‐SCs) with an integrated architecture of electrodes, electrolyte, and separator not only effectively avoid the use of electrochemical‐inactive materials but also significantly reduce the interfacial contact resistances between electrodes and electrolyte.^[^ ^16^ ^]^ The A‐SCs are promising for practical applications because they can avoid any slipping or delamination between electrodes and electrolytes, thereby improving the structural and electrochemical stabilities of devices.^[^ ^17^, ^18^ ^]^ Additionally, A‐SCs could be produced in various shapes and sizes according to complex application requirements, largely increasing the mechanical durability under the compressed, stretched, and twisted states.^[^ ^19^ ^]^ There are two main strategies for the design and construction of A‐SCs. The first is to immobilize electrode materials on the surface of a solid‐state electrolyte through chemical bonds or physical adhesives.^[^ ^20^ ^]^ However, the nondeformable ability of intrinsically rigid electrode materials would produce defects or cracks under repeated deformation–recovery processes, resulting in sharp declines in the performance of A‐SCs. In addition, the construction method usually requires complicated and tedious multiple steps, making it difficult to manufacture devices on a large scale. The second method is to immerse a solid‐state electrolyte directly into the precursor solution with subsequent growth of electrode materials on the electrolyte surface.^[^ ^21^ ^]^ However, it is challenging to grow electrode materials uniformly and controllably on the surface of solid‐state electrolytes. During immersion, the precursors of electrode materials easily diffuse into the electrolyte interior, resulting in a short circuit and reducing the safety of devices. Therefore, it is still challenging to explore new strategies to construct A‐SCs with high stretchability and excellent electrochemical performance.

The conductive polymer is an ideal electrode material for SCs because of its low cost, easy preparation, high theoretical specific capacitance, and intrinsic electrical conductivity.^[^ ^22^ ^]^ As a new‐type hydrogel material, conductive polymer‐based hydrogels have shown both excellent energy storage characteristics of conductive polymers and high stretchability of hydrogels in the potential applications of A‐SCs.^[^ ^23^ ^]^ The superb flexibility of conductive polymer‐based hydrogels allows the assembled A‐SCs to effectively adapt to complex environments.^[^ ^24^ ^]^ In particular, conductive polymer‐based hydrogels could not only provide a continuous ionic conductive path because of their high solvophilic/ionic capacities but also effectively restrain the volume expansion/contraction of electrodes during the charging/discharging processes, thereby endowing the resultant A‐SCs with high‐yet‐stable electrochemical performances.^[^ ^25^ ^]^ However, due to the strong rigidity of the molecular chains of conductive polymers, inevitable agglomerations easily occurred during their in situ polymerization and gelation processes to form hydrogels, significantly reducing the structural stability and conductivity.^[^ ^26^ ^]^ Moreover, the mechanical brittleness of conductive polymer‐based hydrogels is easily caused by structural fractures under complex deformations. Therefore, it is challenging to develop the preparation methods for conductive polymer‐based hydrogels to solve the agglomerations of conductive polymers and meanwhile realize the construction of integrated A‐SCs with deformation‐tolerant performance and electrochemical stability.

In this work, a stretching/cryopolymerization/releasing strategy is proposed to prepare a self‐wrinkled polyaniline (PANI)‐based composite hydrogel (SPCH) film with core–sheath structures, among which an ionic conductive hydrogel (ICH) and an in situ grown PANI‐based composite hydrogel (PCH) are the core and sheath, respectively. The stretching/cryopolymerization/releasing strategy realizes the simultaneous formation of 3D PANI nanostructures and self‐wrinkled surfaces on the ICH electrolyte film. Benefiting from the self‐wrinkled surfaces as well as the abundant hydrogen bonds and electrostatic interactions among the hydrogel film, the resultant SPCH film possessed high mechanical strength (∼0.26 MPa), large stretchability (∼970%), and excellent fatigue resistance (∼100% retention of tensile strength after 1200 cycles). The 3D interconnected PANI avoided the agglomerations of nanoparticles, therefore providing abundant electrochemical active sites. The SPCH also enabled an improved interface between the electrolyte and electrode at the molecular level, optimizing fast ion diffusion kinetics during the subsequent electrochemical reactions. Upon the cutting of edge connections, the SPCH could readily work as an intrinsically stretchable A‐SC, demonstrating high specific capacitance of 209 F g^−1^/504 mF cm^−2^, high energy density of 29.2 W h kg^−1^, outstanding electrochemical stability at a large stretchability of 500%, and full‐scale bending of 180°. This study provides a feasible method for the simultaneous formation of 3D nanostructured conductive polymers and self‐wrinkled surfaces on hydrogel‐based electrolytic films for the straightforward construction of highly deformation‐tolerant A‐SCs.

## 2 RESULTS AND DISCUSSION

**FIGURE 1:**
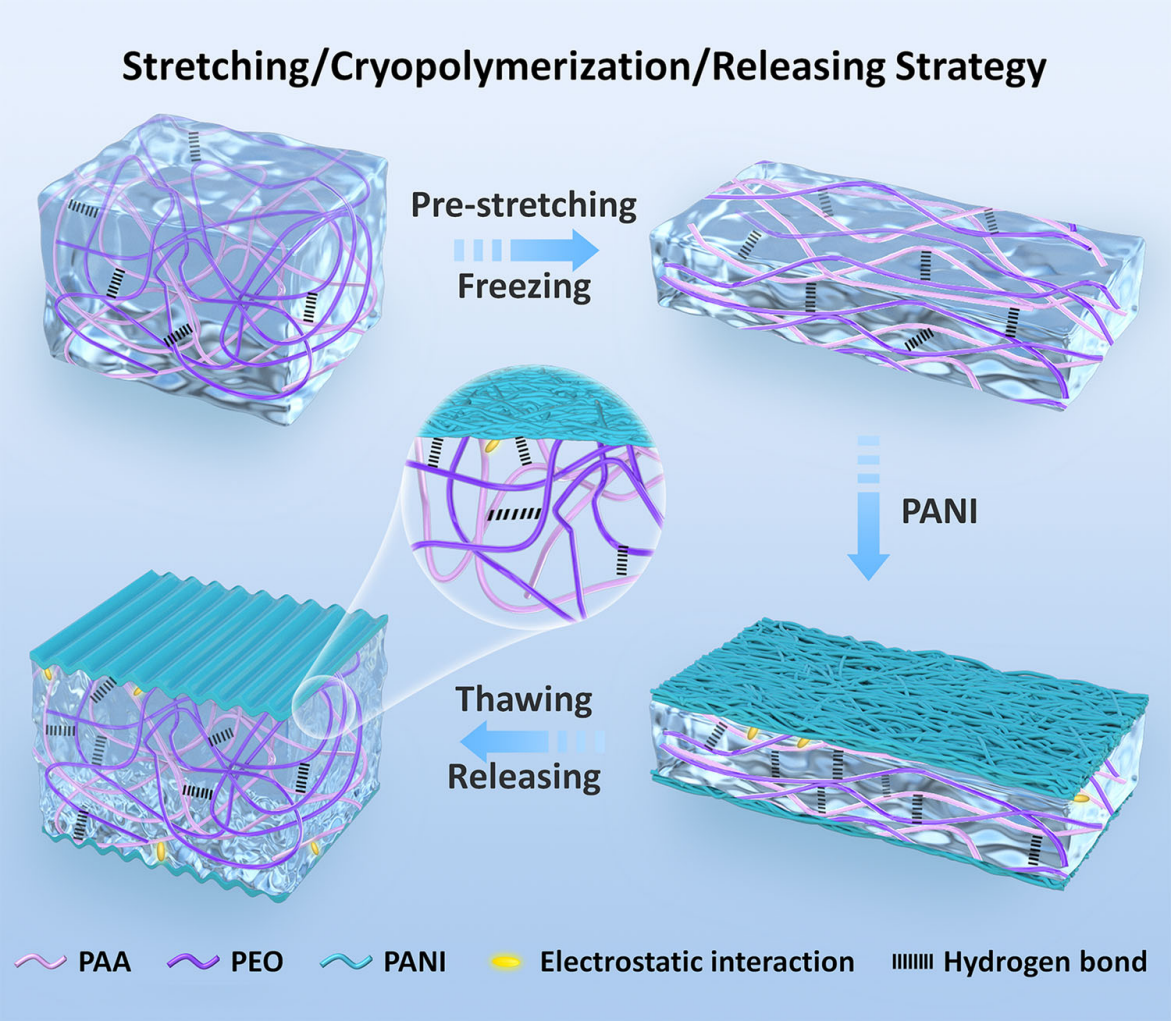
Schematic diagram of the fabrication of SPCH through a stretching/cryopolymerization/releasing strategy

The stretching/cryopolymerization/releasing strategy is proposed for the fabrication of a highly stretchable A‐SC with self‐wrinkled surface structures. To design a highly stretchable A‐SC based on conductive polymers as electrode materials with stable electrochemical performances, especially under complex deformations, our idea is to combine inherent stretchability with wrinkled surface structures to fabricate a self‐wrinkled conductive polymer‐based composite hydrogel. Our previous study indicates that the stretching/competitively coordinating/releasing strategy is suitable for the fabrication of ICHs with wrinkled surface structures.^[^ ^27^ ^]^ Another inspiration is the novel dynamic stretching‐electroplating method for the preparation of metal‐coated textile electrodes with high stretchability and stable conductivity in A‐SCs.^[^ ^28^ ^]^ The authors fabricated a sodium carboxymethyl cellulose‐polyacrylic acid‐potassium hydroxide composite hydrogel as the electrolyte, which showed excellent ionic conductivity, mechanical properties, and water retention properties. A flexible and stretchable sandwich‐structure zinc–air battery was assembled, which could operate stably even under rapid stretching/releasing cycle deformation. In this study, we proposed a new stretching/cryopolymerization/releasing strategy to fabricate a SPCH with an ICH and a PCH as the core and sheath (Figure 1), respectively. A representative conductive polymer of PANI has attracted widespread attention in the use of electrode materials in SCs because of the advantages of high pseudocapacitance, intrinsic electrical conductivity, easy synthesis, and high environmental stability.^[^ ^29^ ^]^ To develop an inherently stretchable A‐SC with an electrode–electrolyte–electrode sandwiched configuration, PANI layers of PCH were in situ polymerized on the surface of the ICH electrolyte film by a stretching/cryopolymerization/releasing strategy. In this study, the initial ICH electrolyte was stretched at specific strains, frozen in liquid nitrogen, and then immersed into a water/ethanol mixture containing ANI monomers and initiators. Through the subsequent cryopolymerization, 3D PANI nanostructures were gradually grown on the frozen ICH surface, forming a closely interacting PCH as the sheath. After thawing and releasing the pre‐stretching tensile force, self‐wrinkled surfaces with permanent shapes were obtained by driving the modulus differences between the relatively rigid PCH sheath and the chemically cross‐linked elastic networks of the ICH core. The research method in this study is beneficial to the preparation of hydrogel materials with complex shape and structure, and it also has positive significance in broadening synthetic methods and applications.^[^ ^30^ ^]^

**FIGURE 2:**
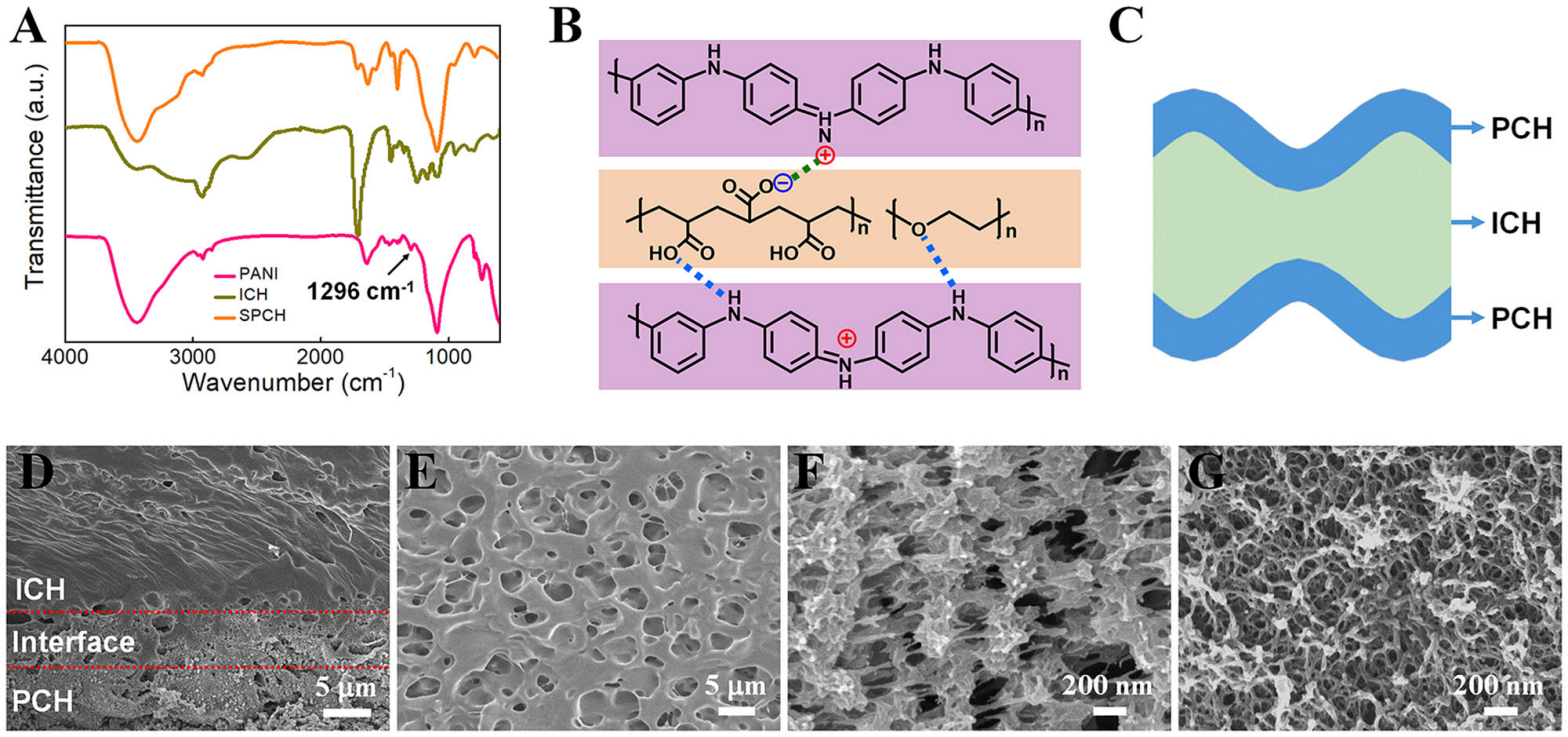
(A) FTIR spectra of PANI, ICH, and SPCH. (B) Schematic of the formation of hydrogen bonds and electrostatic interactions in SPCH. (C) Schematic of the configuration in SPCH. Cross‐section SEM images of (D) full range of SPCH, (E) ICH core, (F) interface layer, and (G) PCH sheath

Upon the cryopolymerization of PANI on the surface of ICH, characteristic bands of PANI appeared (Figure 2A), including the N─H stretching vibration at 3438 cm^−1^, the C═C stretching vibration at 1633 cm^−1^, and the benzene ring vibration at 1091 cm^−1^, proving the evidence of the successful formation of PANI.^[^ ^17^, ^31^, ^32^ ^]^ The characteristic peak of C─N at 1296 cm^−1^ disappeared, which could be explained by the formation of electrostatic and hydrogen bond interactions between the PCH sheath and the carboxyl groups of the ICH core (Figure 2B). The Raman peaks located at 1156, 1212, 1460, and 1583 cm^−1^ are associated with PANI in the SPCH, further demonstrating the successful formation of PANI (Figure S7). Abundant hydrogen bonds, electrostatic interactions, and self‐wrinkled surfaces endowed SPCH with high mechanical strength, large toughness, and excellent fatigue‐resistant performance.

The SPCH with a multilayer structure was obtained (Figure 2C), which was well verified by cross‐section SEM observations. The cross‐section SEM image of SPCH reveals a highly integrated layered structure (Figure 2D), which further indicates the successful formation of the PCH sheath. A clear interface between the PCH sheath and the ICH core was observed (Figure S8). There are no crevices at the interface region, indicating that 3D PANI nanostructures are tightly bonded on the surface of ICH through dense hydrogen bonds and electrostatic interactions.^[^ ^33^, ^34^ ^]^ The lyophilized ICH core presents a porous 3D interconnected network morphology (Figure 2E). The numerous macroporous structures act as a reservoir for the storage of electrolytic ions.^[^ ^35^, ^36^, ^37^ ^]^ Ions in electrolytes could move freely in the 3D hydrogel network without decreasing ionic conductivity. The interface layer depicts the appearance of mixed lamellar‐like and fibrous‐like structures (Figure 2F), which differs from the fibrous PANI layer on the surface (Figure 2G). The differences in the morphology might be attributed to the amalgamation of PANI and ICH along the surface of ICH during cryopolymerization. PANI in the PCH sheath exhibits a 3D nanofibrous porous structure, which avoids the agglomeration of nanoparticles and provides an improved conductive pathway and abundant electrochemical active sites.^[^ ^38^, ^39^ ^]^ A PCH sheath composed of a PANI layer and interface layer is regarded as an electrode layer, and the ICH core is considered an electrolyte layer, thus forming an all‐in‐one configuration of a PCH electrode sheath and ICH electrolyte core. The unique structural design of the SPCH for A‐SCs not only reduces the electrode/electrolyte interfacial resistances but also avoids easy slippage between electrodes and electrolytes in conventional SCs during complex deformations.

**FIGURE 3:**
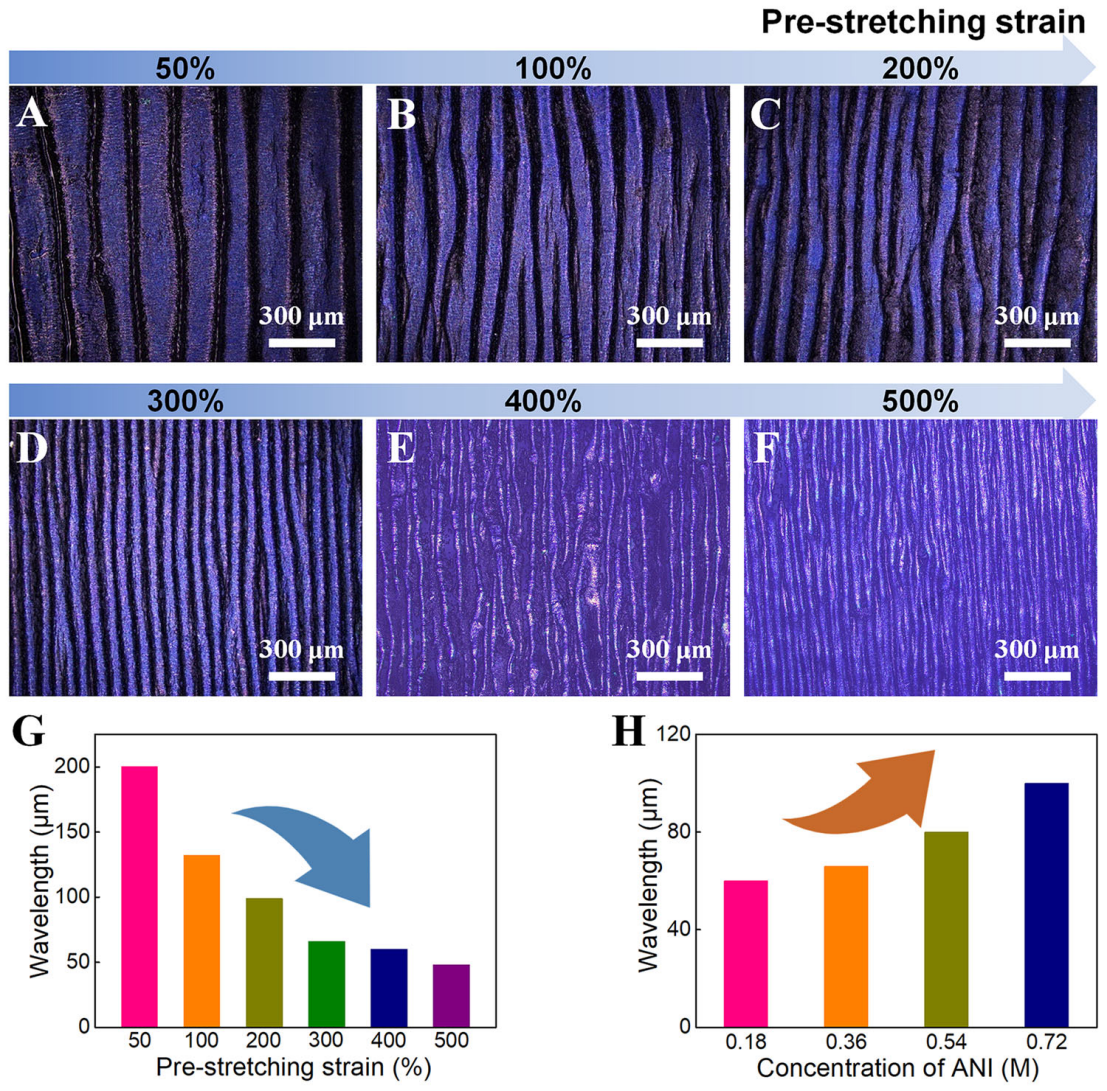
Surface wrinkling morphologies of the SPCHs. (A–F) Surface optical images of the SPCHs prepared under various pre‐stretching strains. Regulations of wavelengths of surface wrinkles by tailoring (G) pre‐stretching strains and (H) ANI concentrations

The surface self‐wrinkled morphology of SPCH was further studied by optical microscopy. The surfaces of the SPCHs display uniform and orderly wavy‐wrinkled structures along the pre‐stretching direction due to the surface immobilization of the PANI structures (Figure 3A–F and Figure S9). The different moduli of the adjacent sheath and core lead to the formation of surface wrinkles in the strain restoration.^[^ ^40^ ^]^ The pre‐stretching strain and aniline (ANI) concentration affected the modulus differences by affecting the initial strain of ICH and shell thickness of PCH and then affected the wrinkled structures. To explain the effect of pre‐stretching strains on the wrinkling structures, the wrinkling wavelengths of SPCHs that were prepared under various pre‐stretching strains were investigated. The self‐wrinkled hydrogel films prepared at various pre‐stretching strains show different wrinkling distances. With the increase in pre‐stretching strains, the wrinkles become more intensive and frequent, and the wrinkled wavelength decreases significantly (Figure 3G).

The characteristics of wrinkles were also modulated by ANI concentrations. The wrinkles become sparse, and the wrinkled wavelength increases significantly with increased ANI concentrations (Figure S9 and Figure 3H). The differences in wrinkle wavelength are caused by the differences in the sheath modulus of elastic SPCH resulting from the different pre‐stretching strains and ANI concentrations.^[^ ^41^, ^42^ ^]^ The more extensive the pre‐stretching strain is, the more compact and ordered PANI nanostructures are formed, resulting in a larger sheath modulus of elastic hydrogels. The thicknesses of the rigid PCH sheaths increase with increasing ANI concentration, which also increases the surface modulus (Figure S10 and Table S3). Consequently, the pre‐stretching strains and ANI concentrations have dominating influences on the wrinkling structures. By changing the pre‐stretching strains and ANI concentrations, the wrinkle wavelengths on the SPCH surface are easily adjusted to adapt to diverse applications.

**FIGURE 4:**
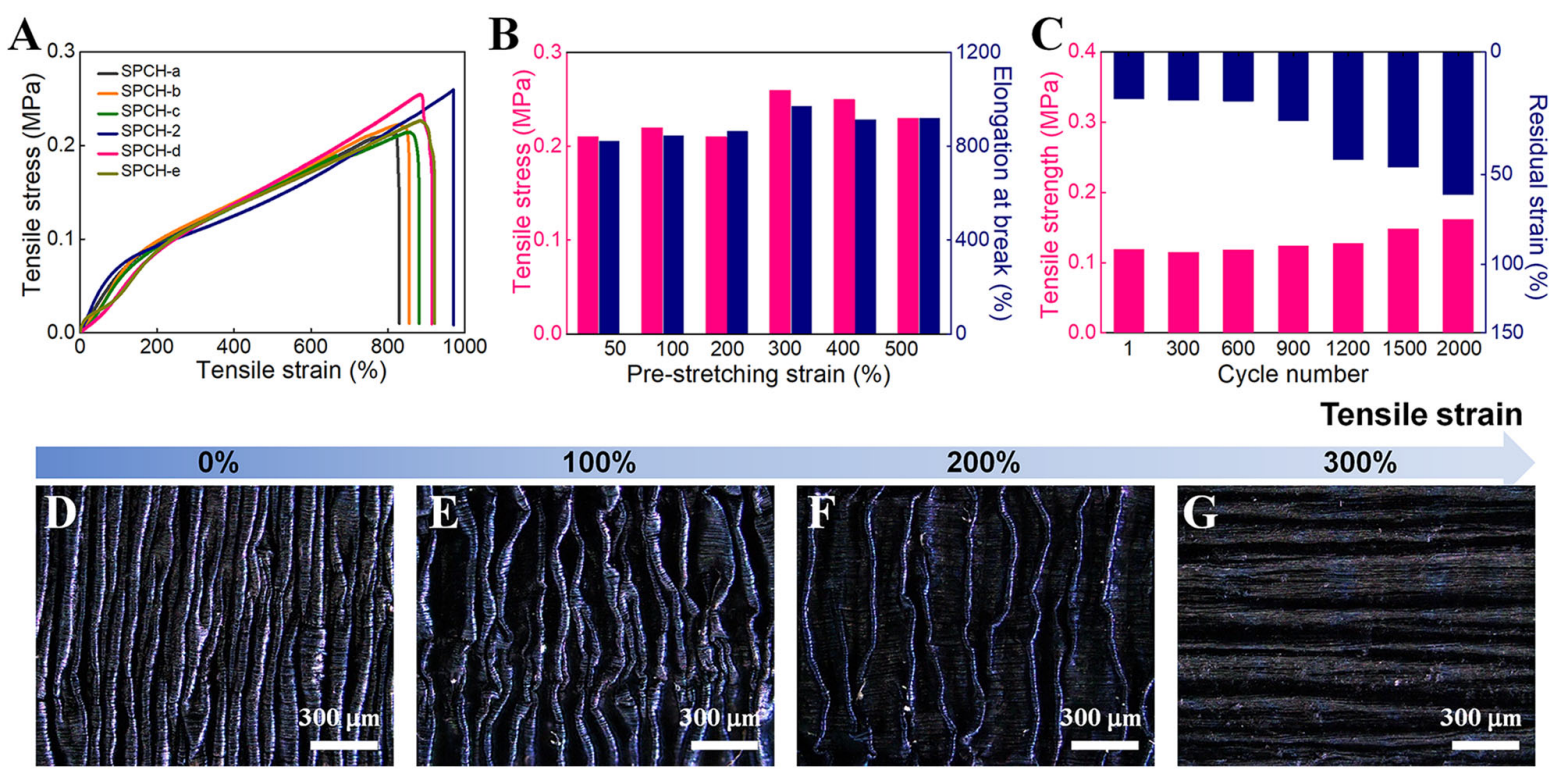
Mechanical properties of the SPCHs. (A) Typical tensile stress–strain curves of the SPCHs fabricated with various pre‐stretching strains. (B) Corresponding tensile stress and elongation at break of the SPCHs from (A). (C) Tensile strength and residual strain of SPCH‐2 after various tensioning–releasing cycles at 200% strain. Surface optical images of SPCH‐2 under the tensile strain of (D) 0%, (E) 100%, (F) 200%, and (G) 300%, respectively

The mechanical properties of SPCH under various fabrication conditions with tailored ANI concentrations and pre‐tensile strains were quantitatively investigated by a series of mechanical performance tests. The SPCHs were easily stretched to a considerable strain of >800% without losing excellent contact between the PCH and ICH (Figure 4A and Figure S11), indicating their excellent structural stability when they were highly stretched. The tensile stress and strain of the SPCHs increase with increasing pre‐stretching strain until SPCH‐2 reaches a maximum tensile strain of 970% and a maximum mechanical stress of 0.26 MPa and decreases with a further increase in the pre‐stretching strain (Figure 4B). This explains why ICH is always in the stretched state under prestrain when PANI is cryopolymerized on the surface. The effect of external forces for a long time would inevitably cause structural defects in ICH, resulting in slight decreases in the tensile stress and elongation at the break of SPCHs prepared under a large prestrain of more than 300%.

According to the thermogravimetric analysis (TGA) results, the mass ratios of PANI in the SPCHs were 1.4 wt% (SPCH‐1), 2.6 wt% (SPCH‐2), 3.4 wt% (SPCH‐3), and 3.6 wt% (SPCH‐4) (Figure S12 and Table S3). The effects of initial ANI concentrations on the mechanical properties of SPCH were also studied (Figure S13A,B). All samples have high tensile stress and elongation at break, possibly ascribing to the unique strengthening effect of the rigid PCH sheath on the surface. The destruction of electrostatic and hydrogen bond interactions between the PCH sheath and the ICH core also contributes to the efficient energy dissipation during stretching. However, elongations at the break of SPCH decrease slightly with the increase of ANI concentrations because the high contents of PANI on the SPCH surface result in the reduction of loading transfer from the ICH core to the PANI nanostructures, leading to the decrease in elongations at the break. Moreover, the high ANI concentrations tend to form a thick rigid PCH sheath, which is more likely to produce structural defects during tensile testing and lead to stress concentration and fracture of SPCH. These results show that the SPCH samples possess excellent mechanical strength and long fracture strain. Unless otherwise stated, SPCH‐2 with an ANI concentration of 0.36 M and a pre‐stretching strain of 300% is selected for subsequent characterizations in the following sections.

Continuous stretching–releasing tests of SPCH‐2 were conducted to observe the fatigue resistance (Figure 4C and Figure S13C). After 1200 stretching–releasing cycles under 200% strain, the tensile strength of SPCH‐2 remains at 100% of its initial value. The mechanical strength of SPCH‐2 increases slightly after 2000 cycles due to the unavoidable evaporation of water during the long‐term fatigue test. After 600 stretching–releasing cycles, the residual strain of SPCH‐2 remains at the value of the first cycle until it increases to 60% after 2000 successive stretching–releasing cycles, exhibiting outstanding mechanical self‐recovery and fatigue resistance. After 2000 stretching–releasing cycles, the self‐wrinkled structure was slightly deformed due to repeated deformation and recovery, while the PCH and ICH remained in excellent contact with each other (Figure S14). The excellent fatigue‐resistant features of SPCH are attributed to the formation of abundant hydrogen bonds among the ICH, the strong interfacial interactions between the ICH core and PCH sheath, and the construction of self‐wrinkled structures. Compared with other conductive polymer‐based composite hydrogels, SPCH‐2 not only has relatively high tensile strength/elongation at break but also possesses superior fatigue resistance (Table S4).

The excellent stretchability and fatigue resistance of SPCH depend mainly on the wavelengths of the repetitive wavy unit.^[^ ^43^ ^]^ The changes in wrinkled structure on the SPCH surface were observed by an optical microscope during the tensile process (Figure 4D–G). The wrinkled structures of the original SPCH are tightly aligned perpendicular to the pre‐stretching direction. With the increase of tensile strain, the wrinkles on the SPCH surface become sparse until they disappear when the strain reaches 300%. During the stretching process, the wavy PCH sheaths are stretched by shape changes at the macroscopic level, while there are no stretching components at the microscopic level. Therefore, the SPCH‐2 prepared under 300% pre‐stretching strain would not cause ruptures or peels off of PCH sheaths when being stretched under the 300% strain, showing its stable integrated structure.

**FIGURE 5:**
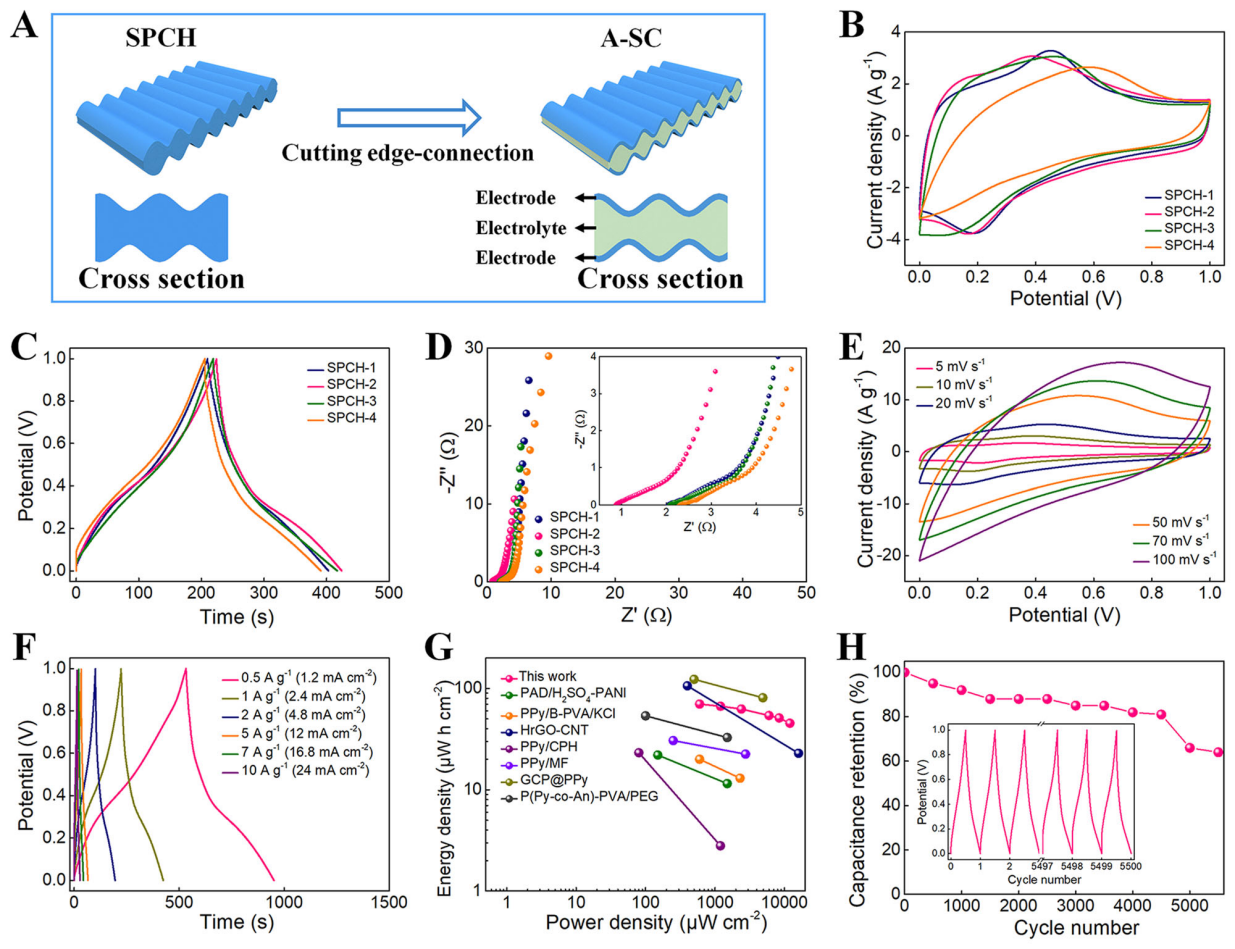
Electrochemical performances of A‐SCs fabricated with the SPCHs. (A) Schematic for the fabrication of the A‐SC. (B) CV (10 mV s^–1^), (C) GCD (1 A g^–1^) curves, and (D) Nyquist plots of A‐SC prepared with various SPCHs. (E) CV curves of SPCH‐2‐based A‐SC at various scanning rates. (F) GCD curves of SPCH‐2‐based A‐SC at various current densities. (G) Ragone plots of SPCH‐2‐based A‐SC in comparison to other A‐SCs in the literature. (H) Cycling performance of SPCH‐2‐based A‐SC at 5 A g^−1^ for 5500 cycles

To design and develop an intrinsically stretchable A‐SC with the electrode–electrolyte–electrode configuration, the edges of a piece of SPCH film were cut off (Figure 5A). The electrochemical properties of the A‐SC devices were evaluated. To obtain the A‐SC with optimized performance, various experiments were carried out to quantitatively investigate the electrochemical performance of SPCHs prepared at different initial ANI concentrations. All the CV curves show significant characteristic redox peaks of PANI (Figure 5B), indicating the Faraday process.^[^ ^26^ ^]^ The non‐straight shape of the GCD curves further confirmed the pseudocapacitive behavior (Figure 5C). At the same time, these representative voltage platforms are in good agreement with the peak values observed in the CV curves. The specific capacitances of A‐SC increase with the increased ANI concentration because PANI nanostructures store sufficient ions to make full use of their specific surface areas. However, the specific capacitance does not increase linearly but gradually decreases when the concentration of ANI is greater than 0.36 M, because the thicker PANI layer lengthens the electron transfer path and blocks the ion transport channel. The interfacial properties of A‐SCs were evaluated by measuring the EIS curves (Figure 5D). The absence of semicircles at high frequency suggests rapid ion movement at the ICH electrolyte–PCH electrode interface. SPCH‐2 has the smallest value of *R_ct_*. As the thickness of the PANI sheath increases, the distance between the anode and cathode increases, and thus, the diffusion resistance increases. Nyquist curves in the low‐frequency region are almost perpendicular to the real axis, manifesting the rapid ion diffusion toward the PCH electrode.^[^ ^44^, ^45^ ^]^ The small charge transfer resistance and rapid ion diffusion give SPCH‐2 optimal capacitance among the A‐SCs, corresponding to the CV and GCD results. The low impedance resulted from the all‐in‐one construction of SCs, where the ICH provides high conductivity, low charge transfer, and electrolyte resistance. The well‐wrapped thin PCH electrode also reduces the interfacial contact resistance.

The CV and GCD curves of A‐SCs fabricated from SPCH‐2 at various scanning rates and current densities were recorded to show the detailed capacitive characteristics. As expected, characteristic redox peaks of PANI appear on the CV curves from 0 to 1.0 V at scanning rates of 5–100 mV s^−1^ (Figure 5E). The GCD curves are typical charge–discharge curves when the current density is between 0.5 and 10.0 A g^−1^ (Figure 5F). Specific capacitances of A‐SCs were calculated according to GCD curves concerning the current densities (Figure S15). When the current density is 0.5 A g^−1^, the A‐SC provides a maximum gravimetric capacitance of 210 F g^−1^ and areal capacitance of 504 mF cm^−2^, which is comparable to previously reported A‐SCs in the literature (Table S4).^[^ ^44^, ^46^ ^]^ When the discharging current density is enhanced to 10 A g^−1^, its gravimetric and areal capacitances remain at 135 F g^−1^ and 326 mF cm^−2^, respectively, indicating its outstanding rate capabilities. The maximum energy density is calculated as 70 µW h cm^−2^ at 600 µW cm^−2^ power density (Figure 5G). When the power density increases to 12000 µW cm^−2^, the energy density remains at 45 µW h cm^−2^. These values are also comparable to or better than those of recently reported A‐SCs.^[^ ^10^, ^16^, ^47^, ^48^, ^49^, ^50^, ^51^ ^]^ The charge–discharge cycles were studied by continuous charge–discharge processes. The A‐SCs exhibit outstanding stability after 5000 cycles, maintaining 66% of the initial capacitance (Figure 5H), indicating that the 3D interconnected porous network of PANI could effectively adapt to the redox reaction process of PCH electrodes and thereby reduce the structural damage of the PCH sheath. The high specific capacitance, rate capability, energy density, and electrochemical stability are ascribed to its porous nanostructures, allowing rapid electron transport through conductive networks, unhindered ion diffusion through micrometer channels, and integrated all‐in‐one prototypes for providing low charge transfer resistance.

**FIGURE 6:**
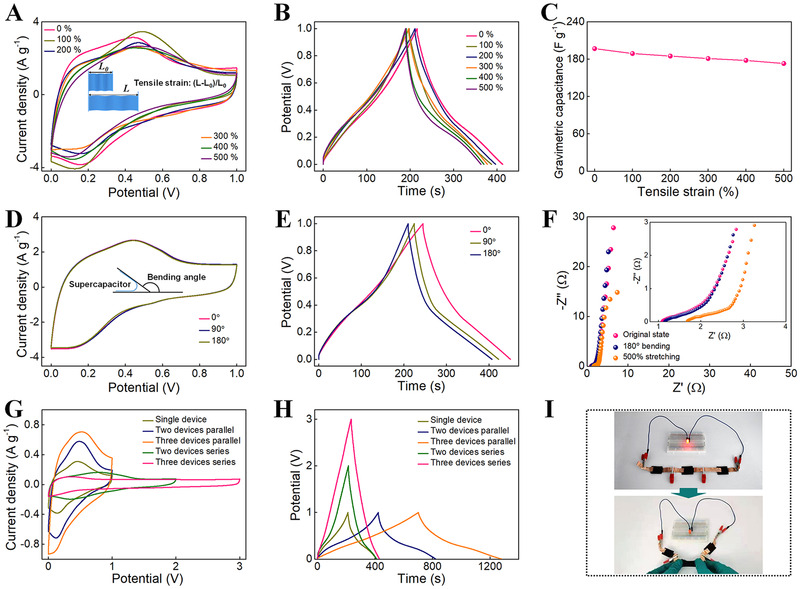
Electrochemical performances of A‐SC from the SPCH under complex deformations. (A) CV curves of A‐SC under various tensile strains at 10 mV s^−1^. Inset of (A) demonstrating the definition of tensile strain. (B) GCD curves of A‐SC under various tensile strains at 1 A g^−1^. (C) Gravimetric capacitances of A‐SC at different tensile strains. (D) CV curves of A‐SC under various bending angles at 10 mV s^−1^. Inset of (D) demonstrating the definition of the bending angle. (E) GCD curves of A‐SC under various bending angles at 1 A g^−1^. (F) Nyquist plots of A‐SC under various deformations. (G) CV (10 mV s^−1^) and (H) GCD (1 A g^−1^) curves of one, two, and three series/parallel‐connected A‐SC devices. (I) Optical photos of an integrated device consisting of three series‐connected A‐SCs lightening up an LED when stretched

Since SPCH has excellent stretchability and high fatigue resistance in mechanical properties, the A‐SC fabricated with SPCH could resist the change in external forces in practical uses, ensuring the stability and safety of power outputs. We confirm the electrochemical stability of A‐SC under mechanical deformations in both static and dynamic modes. The A‐SC shows a high retention ability of gravimetric capacitances under different tensile strains, as demonstrated by CV and GCD curves (Figure 6A–C). When the device is stretched to 300%, the specific capacitance retention is ∼92%. The device could charge and discharge normally even when stretched to 500%, with capacitance retention of >88%. The reduction in specific capacitance is attributed to the increased resistance of ICH electrolytes, which is caused by the loss of junctions in the PCH sheath impeding the motions of electrons and ions. The results indicate that our A‐SC possesses high capacitance retention during the stretching.

Unlike the above tests during deformation, it is also indispensable to evaluate the capacitance performance of A‐SCs after deformation. To confirm the durability, the capacitive performance of the fabricated A‐SC was determined after 1000 stretching–recovering cycles under 100% strain. Areas enclosed by CV curves of the A‐SC still demonstrate large capacitances after 1000 stretching–recovering cycles (Figure S16A). GCD curves further verify that the electrochemical performances are well maintained, and the capacitance retention is approximately 92% (Figure S16B). An increased *Rs* value of the A‐SC device is observed after 1000 cycles of stretching, implying a slight deterioration in electron transport during deformation and resulting in a reduction in capacitance (Figure S16C). Proper deterioration of capacitances and increase of resistances are attributed to the malposition when aligning the cross‐sections to reunite. Considering changes in mechanical properties, the wrinkles on the SPCH surface and the hydrogen bonds/electrostatic interactions among the SPCH affect the electrochemical recovery performance of A‐SC. During deformation, wrinkled structures adapt to the applied strain by changing their shapes. At the same time, the energy dissipation of SPCH is mainly due to the rupture and reconstruction of partial electrostatic and hydrogen bond interactions.^[^ ^52^, ^53^ ^]^ As the outer force is released, the reversible wrinkled structure regains its original shape. The hydrogen bonds and electrostatic interactions as sacrificial bonds could be reconstructed quickly, making the A‐SC quickly restored to its original conductive network structure. The all‐in‐one configuration and intrinsic stretchability helped keep the electrodes close to the electrolyte without delamination and displacement. These factors result in excellent self‐recovery and fatigue resistances of A‐SCs in both mechanical and electrochemical performances.

Due to the inherent stretchability, high mechanical strength, and toughness of the SPCH, the as‐fabricated A‐SC has high bendability. A‐SC shows high retention in gravimetric capacitance under different bending degrees, which can be proven by their almost overlapping CV curves (Figure 6D). The gravimetric capacitance is calculated according to the GCD curves (Figure 6E), reaching more than 95% of its original capacitance when bent at 180°. In addition, EIS measurements were made using Nyquist plots to study electron transport behavior and ion diffusion dynamics at various tensile and bending deformations (Figure 6F). The *R_s_* of A‐SC is almost unchanged when bent at 180°, which is contributed by the close contact between the PCH sheath and ICH core in the bending process. When the A‐SC is stretched to a large strain of 500%, *R_s_* increases significantly, which means that the electron transport slightly deteriorates during deformation. The deformability provides a reliable strategy for building next‐generation long‐life SCs with excellent electrochemical performance and structural integrity.

Our results demonstrate the excellent performance of A‐SC devices prepared by SPCH. To further emphasize the utility of our device, we assembled the A‐SC in series or parallel to evaluate the feasibility of practical application for SPCHs in wearable electronics. The voltage window of the three A‐SCs connected in series is up to 3 V with a similar discharge time, as observed from the CV and GCD curves (Figure 6G,H). A parallel device was also tested by three connected A‐SC devices. Compared to a single A‐SC, the charge and discharge current densities of the integrating device are enlarged three times. The results roughly accord with the basic rule of series and parallel connections of capacitors.^[^ ^51^ ^]^ These results demonstrate the feasibility of scaling up our A‐SCs into wearable devices. An integrated device consisting of three A‐SCs in series could light up an LED at 100% deformation (Figure 6I), demonstrating its outstanding deformation‐tolerant energy storage capability.

For comparison, the A‐SC based on the CPCH was prepared and investigated (Figures S17–S19). The specific capacitance of CPCH‐based A‐SC is 189 F g^−1^, comparable to that of SPCH‐assembled A‐SC (Figure S20). The CV and GCD curves at various scanning rates and current densities show that CPCH‐based A‐SC could withstand a high scanning rate and current density of 100 mV s^−1^ and 10 A g^−1^, respectively (Figure S21A,B), mainly because of the high theoretical specific capacity, high electrical conductivity, and good charging and discharging capacities of conductive PANI.^[^ ^54^ ^]^ The specific capacitance of the device at various current densities is calculated by the GCD curve. The A‐SC device possesses excellent rate performance (Figure S21C), which is slightly reduced compared to the SPCH‐assembled A‐SC. This phenomenon might be due to the continuous embedding of PANI into ICH during the traditional CPCH preparation process, which makes the PANI and ICH blend tightly. During the rapid charge and discharge process, electrolyte ions and electron migration are easily blocked, resulting in fast drops in capacitances at high current densities.

**FIGURE 7:**
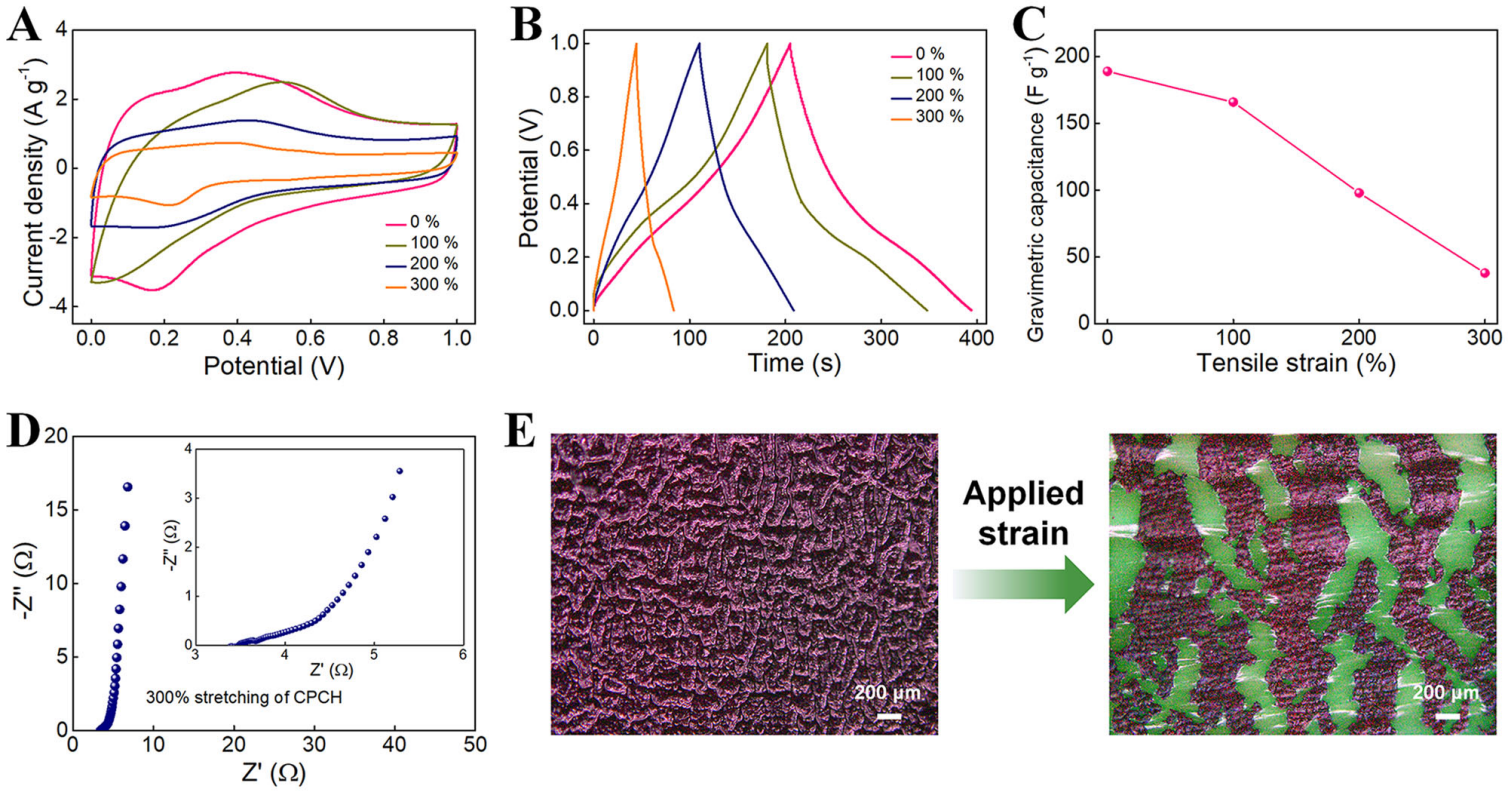
Electrochemical performance of A‐SC based on CPCH. (A) CV (10 mV s^−1^), (B) GCD (1 A g^−1^) curves, and (C) gravimetric capacitances of A‐SC under various tensile strains. (D) Nyquist plot of the A‐SC at 300% tensile strain. (E) Surface optical images of the CPCH at the initial and stretching (300% strain) states

When the A‐SC device is stretched, the corresponding capacitances decline significantly, dropping quickly compared with the SPCH‐based A‐SC (Figure 7A–C). When the A‐SC device is stretched to 300%, the specific capacitance is only 38 F g^−1^. If the tensile strain continues to increase, the device cannot be charged and discharged properly. The results indicate that the electrochemical performance of A‐SC based on CPCH degrades rapidly under large tensile deformation, which is not conducive to its operation in complex deformation environments. Furthermore, based on the Nyquist plots (Figure 7D), it can be seen that the *R_s_* of CPCH‐based A‐SC is much larger than that of SPCH‐based A‐SC in the low‐frequency region, suggesting a significant increase in the contact resistances. The rapid reduction in specific capacitances and the significant increase in contact resistances are caused by the rupture of the rigid PANI electrode material during stretching (Figure 7E). In addition, the uneven distribution of PANI is unfavorable for effective charge transfer and ion diffusion between individual PANI nanoparticles.

According to the above analysis, the stretching/cryopolymerization/releasing strategy not only realizes the controllable and uniform growth of 3D PANI nanostructures on the ICH core but also constructs the self‐wrinkled surfaces of PCH, thereby realizing the construction of an all‐in‐one integrated structure of SCs. Intrinsically stretchable A‐SCs overcome the inconvenience of device assembly in a large‐scale area caused by multilayer structures. As a result, the SPCH could be fabricated into high‐energy‐density storage devices with high stretchability for wearable applications. Our research might pave the way for developing self‐wrinkled conductive polymer‐based composite hydrogels for A‐SCs with high deformation tolerance and high‐energy‐density energy storage.

## 3 CONCLUSION

In conclusion, we have presented a stretching/cryopolymerization/releasing strategy for preparing an SPCH film with the PCH layer and ICH layer as sheath and core, respectively. The stretching/cryopolymerization/releasing strategy provides a simple and efficient approach for in situ growing PCH layer with 3D interconnected PANI nanostructures on the surface of the ICH film. Benefiting from the macroscopic self‐wrinkled structures and microscopic dynamic reversible interactions of hydrogen bonds and electrostatic interactions, the resultant SPCH exhibited high stretchability of ∼970%, excellent ultimate mechanical strength of ∼0.26 MPa, and super fatigue resistance for 1200 stretching–releasing cycles at 200% strain. Due to the tailored all‐in‐one configuration and self‐wrinkled surface, the as‐obtained SPCH could work as an intrinsically stretchable A‐SC, which could maintain high specific capacitances of 504 mF cm^−2^ and 210 F g^−1^ at 0.5 A g^−1^, high energy density of 70 µW h cm^−2^, and outstanding cycling stability of 5000 charge–discharge processes. The A‐SC could also deliver stable electrochemical properties under large deformations of 500% tensile strain and fully bending of 180°, and the capacitance retention could reach 92% after 1000 consecutive stretching–recovering cycles at 100% strain. This study might pave the way for developing conductive polymer‐based hydrogels with self‐wrinkled surfaces for all‐in‐one stretchable supercapacitors with large deformation tolerance and high energy density.

## 4 EXPERIMENTAL SECTION

Experimental details are provided in the Supporting Information.

## CONFLICT OF INTEREST

The authors declare no conflict of interest.

## Supporting information

Supporting Information

## Data Availability

The data that support the findings of this study are available from the corresponding author upon reasonable request.
