# Supplementary material for: Cryopolymerization‐enabled self‐wrinkled polyaniline‐based hydrogels for highly stretchable all‐in‐one supercapacitors
Source: Exploration (Beijing). 2022 Jul 4;2(4):20220006. doi: 10.1002/EXP.20220006 (PMC10191047; doi:10.1002/EXP.20220006)
Supplement: Supplementary file 1 — Supporting Information [file EXP2-2-20220006-s001.docx]

Supporting Information

**Cryopolymerization-enabled self-wrinkled polyaniline-based hydrogels for highly stretchable all-in-one supercapacitors**

Hui Song^1^, Yufeng Wang^1^, Qingyang Fei^1^, Dai Hai Nguyen^3^, Chao Zhang^1^*, Tianxi Liu^1,2^*

^1^*State Key Laboratory for Modification of Chemical Fibers and Polymer Materials, College of Materials Science and Engineering, Donghua University, Shanghai, P.R. China*

^2^*Key Laboratory of Synthetic and Biological Colloids, Ministry of Education, School of Chemical and Material Engineering, International Joint Research Laboratory for Nano Energy Composites, Jiangnan University, Wuxi, P.R. China*

^3^*Institute of Applied Materials Science, Vietnam Academy of Science and Technology, Ho Chi Minh City, Vietnam*

**S1. Materials**

Polyethylene oxide (PEO, average *M_v_* = 600000), sodium hydroxide (NaOH, AR, ≥ 96%) and *N*, *N’*-methylene-bisacrylamide (MBAA, 96%) were obtained from Aladdin Chemicals. Acrylic acid (AA, AR, ≥ 99.0%) was obtained from Adamas. Aniline (ANI, AR, ≥ 99.5%), ammonium persulfate (APS, AR, ≥ 98%), sulfuric acid (H_2_SO_4_, 98%), and hydrochloric acid (HCl, 36-38%) were obtained from Sinopharm Chemicals. All the chemicals were used without further purifications. Deionized (DI) water was used throughout the experiments.

**S2. Preparation of ICH**

PEO solution was prepared by dissolving a designed amount of PEO in water. Designed amounts of NaOH (n(AA)/n(NaOH): 10/1), MBAA (0.5 wt % of AA monomers), AA, and APS (1 wt % of AA monomers) were added into the PEO solution under stirring. The mixed solution was rapidly degassed in a vacuum, cast into a polytetrafluoroethylene mold, and subsequently heated at 70 °C to initiate polymerization for 12 h. The PEO-PANa hydrogel was immersed into an aqueous solution of 1 M H_2_SO_4_ for 1 h and aged for another 12 h to obtain ionic conductive hydrogels (ICHs). The ICH-1, ICH-2, ICH-3, ICH-4, and ICH-5 represent the resultant ICHs samples that were prepared at the molar ratio of AA and EO (hereafter, the molar ratio of repeating units shown as [AA]/[EO]) of 1.6/1, 1.3/1, 1/1, 1/1.3, and 1/1.6, respectively (details listed in Table S1).

**S3. Preparation of self-wrinkled polyaniline (PANI)-based composite hydrogel (SPCH)**

Designed amounts of ANI, APS, and HCl were added into the mixture of water and ethanol (*v*(H_2_O)/*v*(ethanol): 1/1) under stirring at -25 °C. Subsequently, pre-stretched strips of ICH were frozen in liquid nitrogen and immersed in the mixed solution. The frozen samples were maintained at -25 °C for 3 h to achieve the cryopolymerization of ANI. Upon thawing, the SPCHs were obtained by washing with excess water to remove oligomers and impurities completely. The as-obtained SPCHs are marked as SPCH-1, SPCH-2, SPCH-3, and SPCH-4 when the initial ANI concentrations of 0.18, 0.36, 0.54, and 0.72 M were used, respectively, while keeping the pre-stretched strain at 300%. Besides, the SPCH-a, SPCH-b, SPCH-c, SPCH-d, and SPCH-e represent the ICH samples that were pre-stretched to the strain of 50%, 100%, 200%, 400%, and 500%, respectively, with the initial ANI concentration of 0.36 M. The molar ratio of ANI/APS was kept at 2, while the concentration of HCl was kept at 1 M. Conventional PANI-based composite hydrogel (CPCH) was prepared by soaking the unstretched ICH in ANI solution with subsequent cryopolymerization for 1 h, while the other conditions were the same as that for the SPCH-2 (Table S2).

**S4. Materials characterization**

Chemical compositions and interactions of freeze-dried hydrogels were analyzed by Fourier transform infrared (FTIR, Nicolet 6700) in a scan range of 600-4000 cm^-1^ and Raman spectra on a Dilor LABRAM-1B multi-channel confocal microspectrometer with a 631 nm laser excitation. Side- and top-view images of hydrogel samples were tested by an optical microscope (Olympus BX53M). Microstructures and morphologies of freeze-dried hydrogels were examined by field-emission scanning electron microscopy (FESEM, JSM-7500F) at an acceleration voltage of 5 kV. Weight contents of PANI within freeze-dried SPCH sample were investigated by thermogravimetry (TGA) on a thermal analyzer (TG209F1, NETZSCH, USA). Mechanical properties of hydrogels were measured using a universal testing machine (UTM2102, SUNS). For tensile measurements, rectangular hydrogel samples (2 × 5 × 10 mm^3^) were stretched at a strain rate of 10 mm min^-1^. For compression tests, cylindrical hydrogel samples (diameter: 15 mm, height: 20 mm) were compressed at a speed of 10 mm min^-1^. Ionic conductivity of ICH was studied by electrochemical impedance spectroscopy (EIS) on a CHI 660D electrochemical workstation. The ICH sample was sandwiched between two electrodes, and the ionic conductivity (σ) is calculated as follows:

$\sigma=\frac{L}{R_{b}\times S}$ (1)

where, *L* is the distance between electrodes, *R_b_* is the bulk resistance, and *S* is the contact area between ICH and electrodes.

**S5. Electrochemical measurement of the A-SC based on SPCH**

Edges of a piece of SPCH sample were cut off to avoid a short circuit, and the hydrogel sample was trimmed to the desired dimension (width: 1 cm, length: 2 cm, thickness: 0.2 cm). Subsequently, two carbon paper strips (width: 0.1 cm, length: 2.5 cm, thickness: 0.001 cm) were placed on both sides of the hydrogel sample as current collectors to connect the electrochemical station.

The electrochemical performance of A-SC was evaluated in a two-electrode system with a CHI 660 D workstation. Cyclic voltammetry (CV) tests were performed in the potential range of 0-1 V under a scan rate of 5-100 mV s^-1^. Galvanostatic charge/discharge (GCD) tests were carried out at various current densities from 0.5 to 10 A g^-1^ with a potential range of 0-1 V. EIS measurements were conducted with a frequency range of 10^5^ to 0.01 Hz with a 10 mV amplitude at the open-circuit potential. Cycling stability of A-SC was carried out by repeating the GCD tests at 5 A g^-1^. To avoid the influence of water evaporation during the long-time cycling tests, the prepared A-SC was stored in a polyethylene sealed bag. Tensile-tolerant performance of A-SC was evaluated by measuring CV and GCD curves when the device was stretched. Gravimetric capacitances of A-SC were calculated from GCD curves according to Equation (2):

$C_{s}=I\times t/(M\times V)$ (2)

where *I* is the discharge current, *t* is the discharge time, *M* is the total mass of PANI within two electrodes, and *V* is the voltage change upon discharging.

Energy density and power density of A-SC were obtained based on Equations (3) and (4), respectively:

$E=(1/2)\times C_{s}\times V^{2}$ (3)

$P=E/t$ (4)

where *E, C_s_, V, P,* and *t* are the energy density, gravimetric capacitance of A-SC, voltage change upon discharging, power density of A-SC, and discharge time, respectively.

**S6. Fabrication and structures of ICH**

As shown in Figure S1, hydrogen-bonded crosslinked ICH was prepared by thermal initiated free-radical polymerization. In a typical experiment, the desired PEO-PANa hydrogel was synthesized by free-radical polymerization of AA monomers with MBAA served as a collaborative cross-linking agent in the mixed solution of PEO and NaOH. Subsequently, the PEO-PANa hydrogel was immersed in a sufficient volume of 1 M H_2_SO_4_ solution to protonate PAA and produce ICH. As a result, ICH was stable in an acidic environment and was favorable as an electrolyte material for supercapacitors, which also typically operated in acidic environments. The addition of H_2_SO_4_ endowed the ICH electrolyte with high ionic conductivity, which was measured by EIS (Figure S2). By calculating from the charge transfer resistance (*R_ct_*), the ICH obtained high ionic conductivity of 0.18 S m^-1^, making it promising to act for gel electrolyte material. Dense hydrogen bonds between the PEO and PAA chains were investigated by FTIR. Based on the FTIR spectra of PEO, PAA, and ICH (Figure S3), the peaks at 1348, 1089, and 948 cm^-1^ of ICH were assigned to the vibrations of -CH_2_, C-O-C, and C-H bonds of PEO, respectively.^[1]^ The peaks at 1451, 1248, and 1166 cm^-1^ were attributed to the vibrations of C-O, C-C, and C-H bonds of PAA, respectively.^[2]^ All these characteristic peaks indicated the presence of both PEO and PAA chains in ICH. Compared with the freeze-dried ICH, the C=O stretching vibration at 1698 cm^-1^ in the pristine PAA hydrogel shifted to lower frequencies, verifying the existence of strong hydrogen bonds between the PEO and PAA chains.^[3]^ More importantly, a new and wide absorption peak appeared near 3300-3600 cm^-1^ in ICH, indicating that intermolecular hydrogen bonds were formed between the PAA and PEO chains. The dynamical bonds can dissipate the elastic energy through reversible bond cleavage and reformation, giving the ICH excellent stretchability resilience and favorable mechanical strength.

**S7. Mechanical performance of ICH**

The mechanical properties of ICHs were investigated by both tensile and compressive stress-strain measurements. Tensile stress-strain curves of ICHs prepared with various molar ratios of [AA]/[EO] have been characterized. As the [AA]/[EO] molar ratios decrease, the elongation at break of the ICH electrolytes gradually increases from 716 to 1278%, whereas the ultimate tensile strength significantly reduces from 0.26 to 0.13 MPa, which is much higher than that of PAA hydrogel (Figure S4). Compression stress-strain curves of ICH indicate that the compression stress decreases with the decrease of [AA]/[EO] molar ratios, but it is always higher than that of PAA hydrogel (Figure S5). It is because the PEO molecules possess high flexibility and low mechanical strength.^[3]^ The ICH-3 is selected for the following experiments because of the trade-off between the tensile strength and elongation at break. Anti-fatigue properties of the ICH-3 were evaluated by carrying out multiple loading-unloading tests. After 1000 stretching-releasing cycles, the tensile strength of ICH-3 maintains 92% of its original value, while the residual strain is 19% (Figure S6A). Furthermore, upon 1000 compression-recovery cycles at a 50% strain, the hysteresis loops of the ICH-3 are almost overlapped, indicating its excellent recovering properties and compressive stability (Figure S6B). The high stretchability and compressive resistance of ICH originate from the dense hydrogen bonds. Thus, our hydrogel is highly resistant to constant mechanical damage without interrupting its operation.

**S8. Fabrication of SPCH**

During the fabrication of SPCH, the pre-stretched ICH strip was frozen slowly in liquid nitrogen until the sample was frozen into dense ices. It is noted that the pre-strain is constrained among the frozen strip, and once thawed, the sample would return to its original unstressed shape. However, if the pre-stretched ICH strip was dropped fast into liquid nitrogen for rapid freezing, it would easily get fractures due to the sudden cooling, losing the structural integrity. The freezing in this study is quite different from the traditional ice-templating process. That is, the strip would not be dissolved or swelled in water although the ice crystals would grow into templates during the freezing process. In this study, the as-prepared ICH film has chemically crosslinked networks before freezing, and the restriction of ice crystals would not affect the arrangement of network structures of ICH. Therefore, the cooling method (such as directional freezing or two-way frozen), cooling rate, and cooling medium (such as liquid nitrogen, frozen ethanol bath, or refrigerator) have no substantial effects on the properties of the ICH. The self-wrinkled PANI-based composite hydrogels were obtained by driving the modulus differences between the relatively rigid PCH sheath and the ICH core. The freezing method, rate, and medium did not affect the self-wrinkled structures and surface morphologies of the SPCH.

The frozen pre-stretched ICH was then immersed into the mixed solution of ethanol and water containing ANI monomers and APS for subsequent cryopolymerization at -25 ℃. The presence of H_2_SO_4_ inhibited the growth of ice crystals, endowing ICH with a low-temperature resistance of -25 ℃. The frozen ICH would dissolve when the temperature was above -25 ℃, resulting in the failure preparation of self-wrinkled PANI-based composite hydrogels by the stretching/cryopolymerization/releasing strategy. With the polymerization of PANI, rigid PANI-based composite hydrogel sheaths were formed on a soft ICH core surface. Driven by the modulus difference between the PCH sheath and ICH core, self-wrinkled PANI-based composite hydrogels were obtained after thawing and releasing the pretension.^[7, 8]^


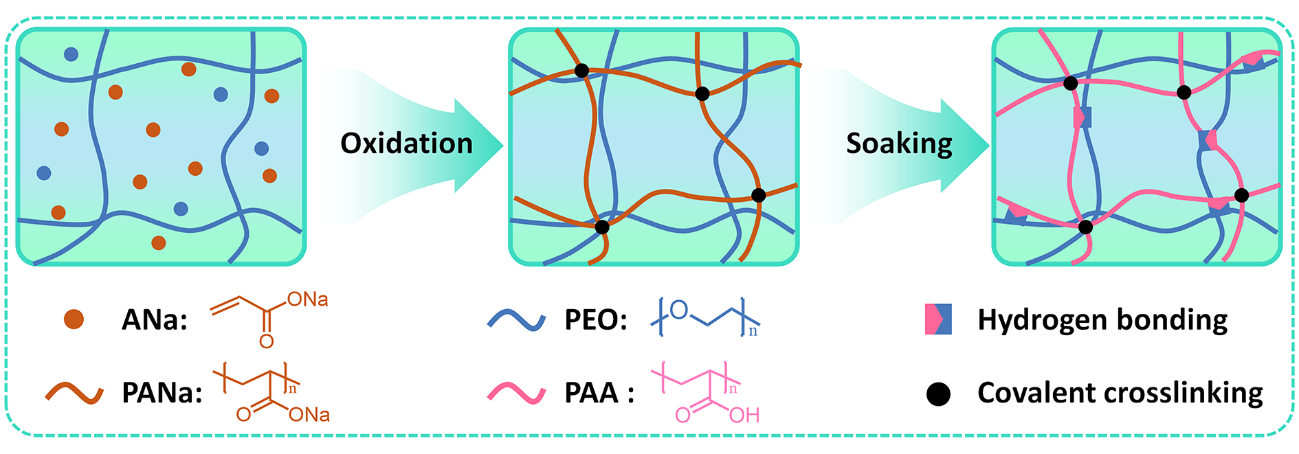


**Figure S1.** Schematic of the fabrication of ICH.


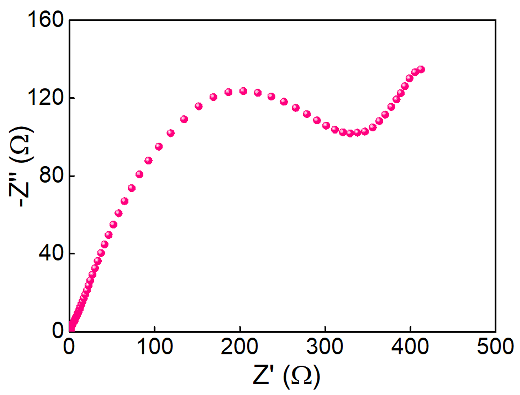


**Figure S2.** Nyquist plot of ICH.


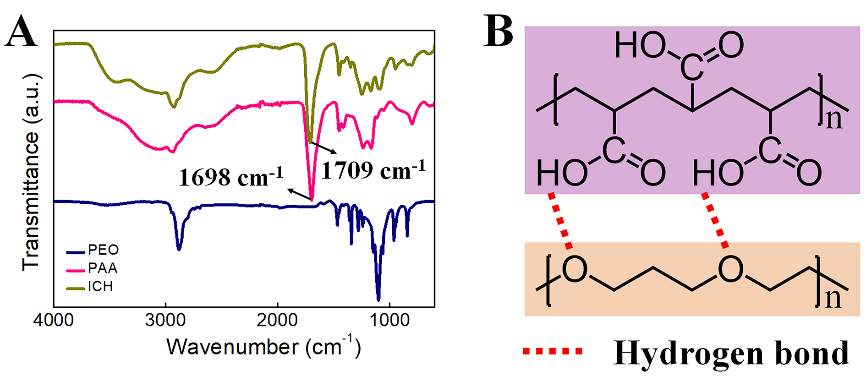


**Figure S3.** (A) FTIR spectra of PEO, PAA, and ICH. (B) Schematic of the hydrogen-bonded interactions between PAA and PEO chains.


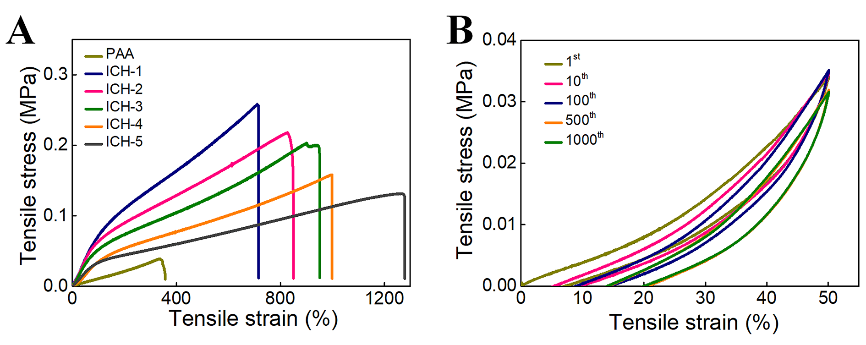


**Figure S4.** (A) Tensile stress-strain curves of PAA and ICH with a variety of [AA]/[EO] molar ratios. (B) Successive stretching-recovering cycles of ICH-3 at a strain of 50%.


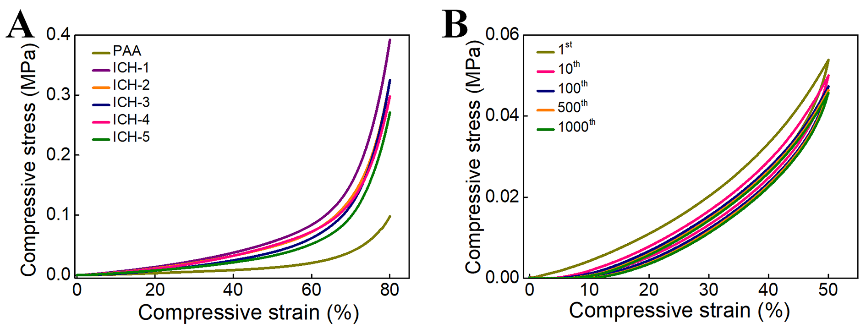


**Figure S5.** (A) Compressive stress-strain curves of PAA and ICH with a variety of [AA]/[EO] molar ratios. (B) Successive compressing-recovering cycles of ICH-3 at a strain of 50%.


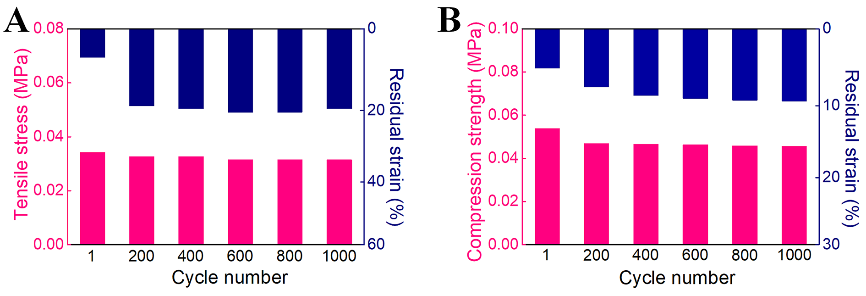


**Figure S6.** (A) Corresponding tensile stress and residual strain of ICH-3 after various cycles at a strain of 50%. (B) Compression strength and residual strain of ICH-3 after various cycles at a strain of 50%.


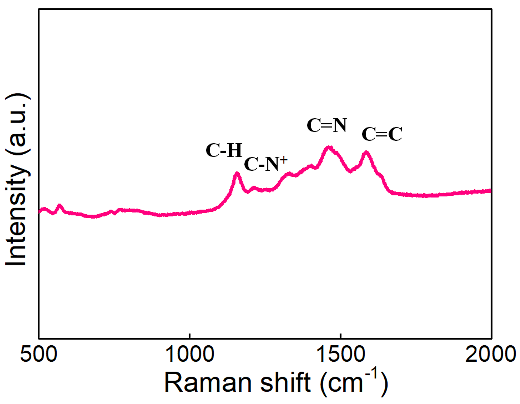


**Figure S7.** Raman spectrum of SPCH


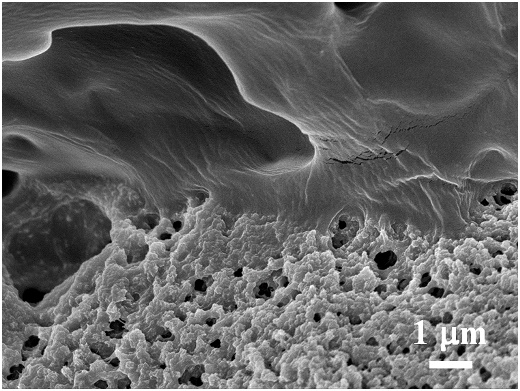


**Figure S8.** SEM image of interfaces at high magnification.


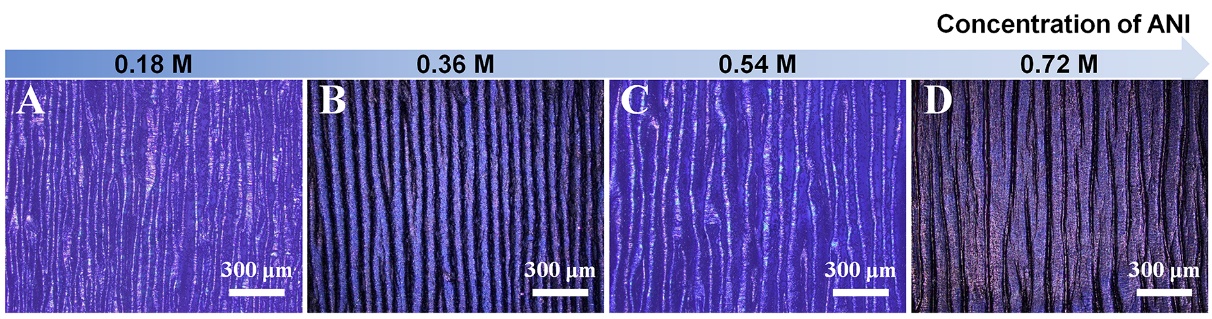


**Figure S9.** Surface optical images of SPCHs prepared under various ANI concentrations.


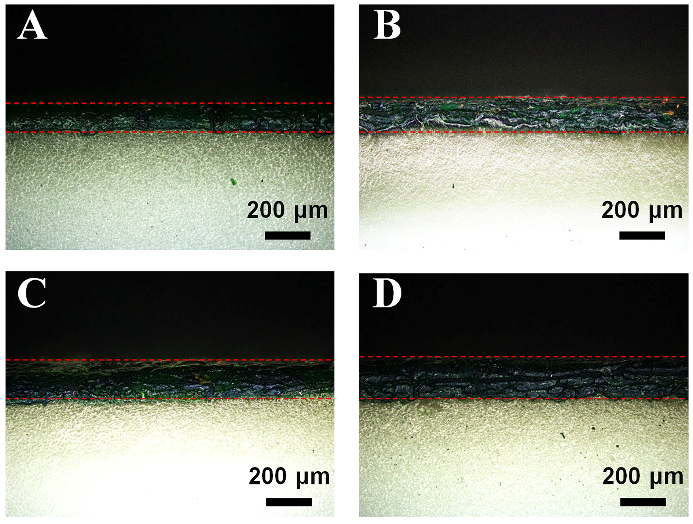


**Figure S10.** Cross-sectional optical images of (A) SPCH-1, (B) SPCH-2, (C) SPCH-3, and (D) SPCH-4.


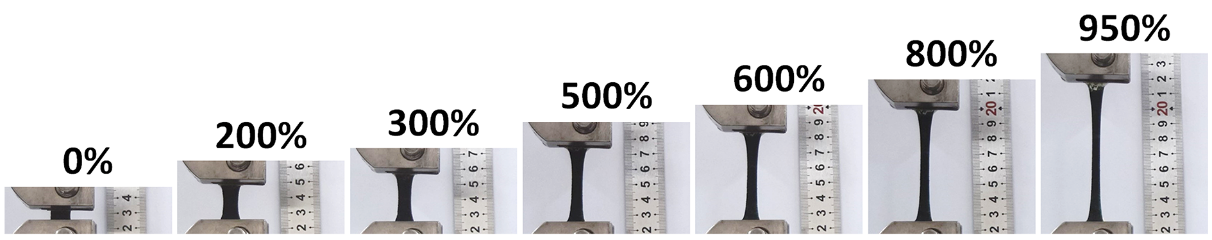


**Figure S11.** SPCH samples stretched at a strain from 0 to 950%.


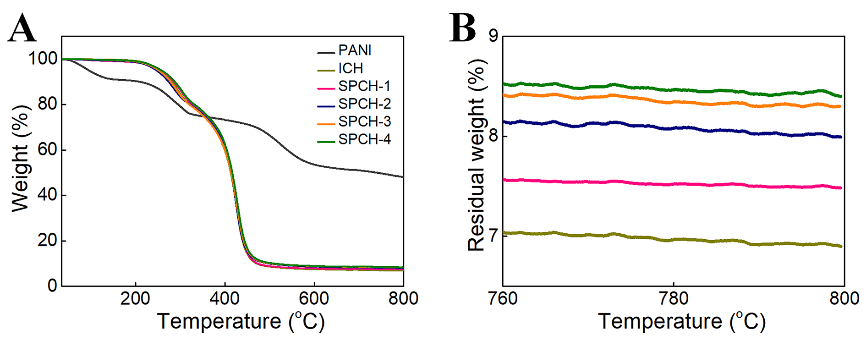


**Figure S12.** (A) TGA curves of PANI, ICH, and SPCH in nitrogen. (B) Enlarged areas of TGA curves within the temperature range of 760-800 °C.


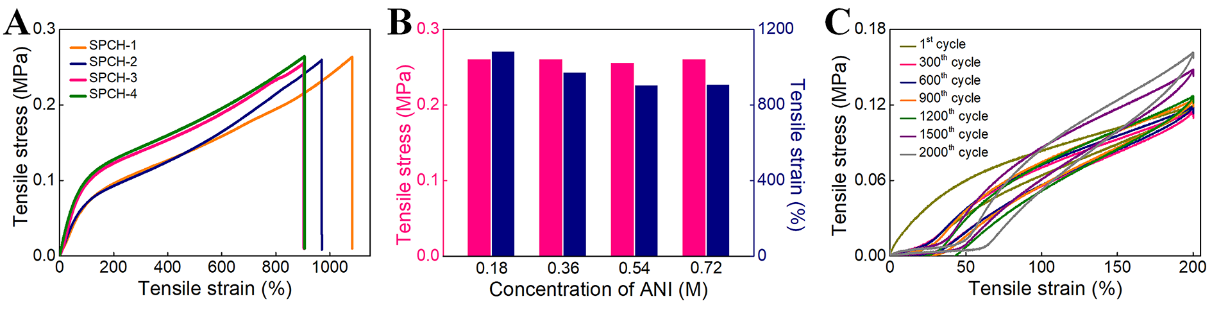


**Figure S13.** (A) Tensile stress-strain curves of SPCHs prepared under various ANI concentrations. (B) Corresponding tensile stress and strain obtained from (A). (C) Successive stretching-recovering cycles of SPCH-2 at a strain of 200%.


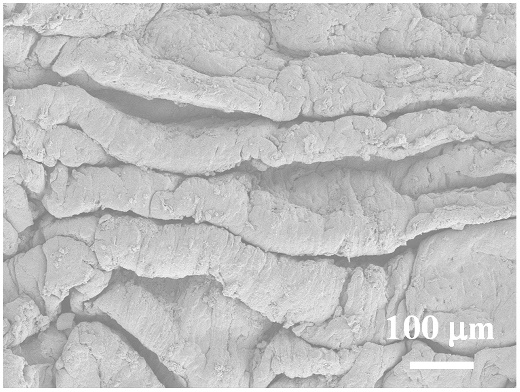


**Figure S14.** SEM image of SPCH after 2000 stretching-releasing cycles under 200% strain.


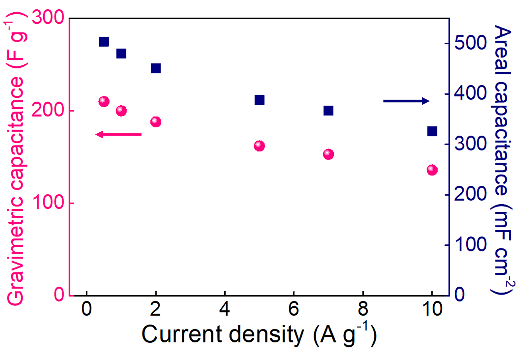


**Figure S15.** Gravimetric and areal capacitances of A-SC prepared from SPCH-2 at various current densities.


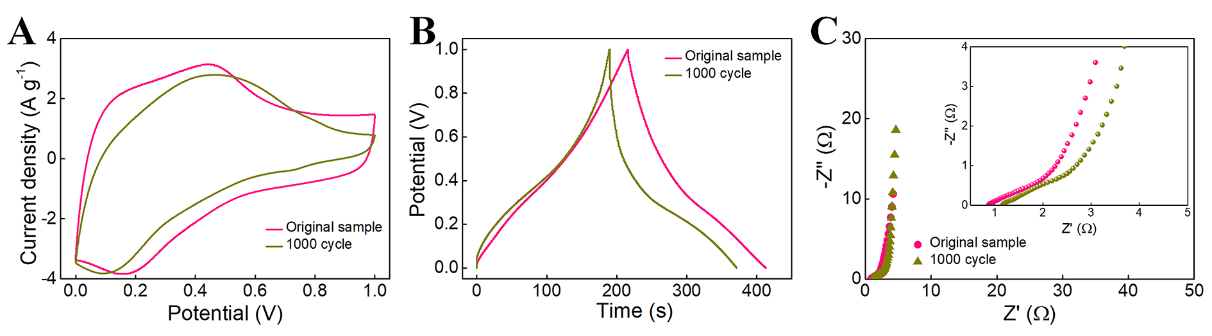


**Figure S16.** (A) CV curves (10 mV s^-1^), (B) GCD curves (1 A g^-1^), and (C) Nyquist plots of the A-SC based on SPCH-2 and SPCH-2 after 1000 successive stretching-recovering cycles at a strain of 100%.


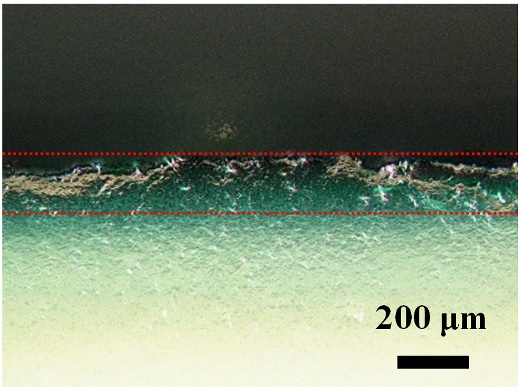


**Figure S17.** Cross-sectional optical image of CPCH.


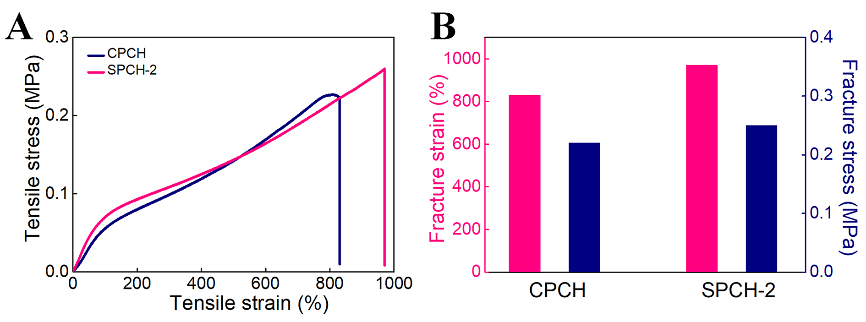


**Figure S18.** (A) Tensile stress-strain curves of CPCH and SPCH-2. (B) Fracture strain and stress of CPCH and SPCH-2.


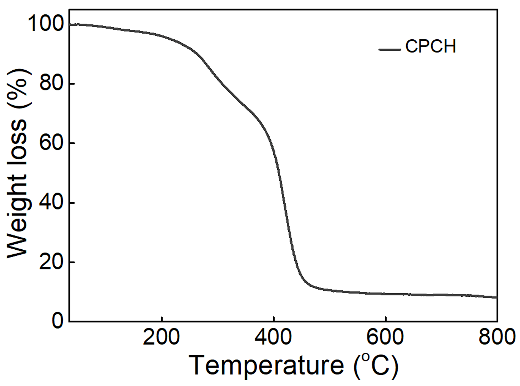


**Figure S19.** TGA curve of CPCH in nitrogen.


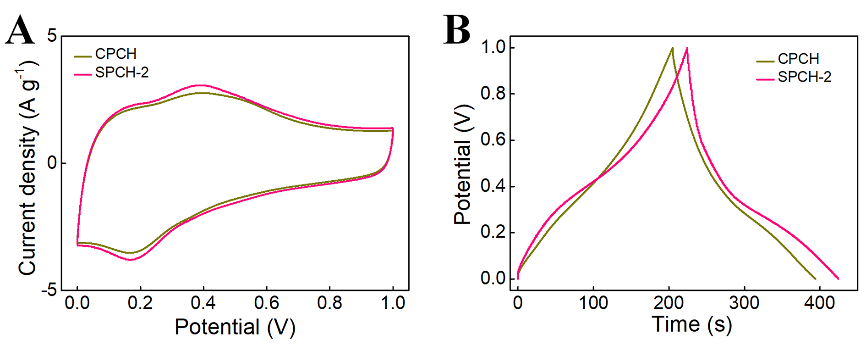


**Figure S20.** (A) CV curves (10 mV s^-1^) and (B) GCD curves (1 A g^-1^) of A-SC based on CPCH and SPCH-2.


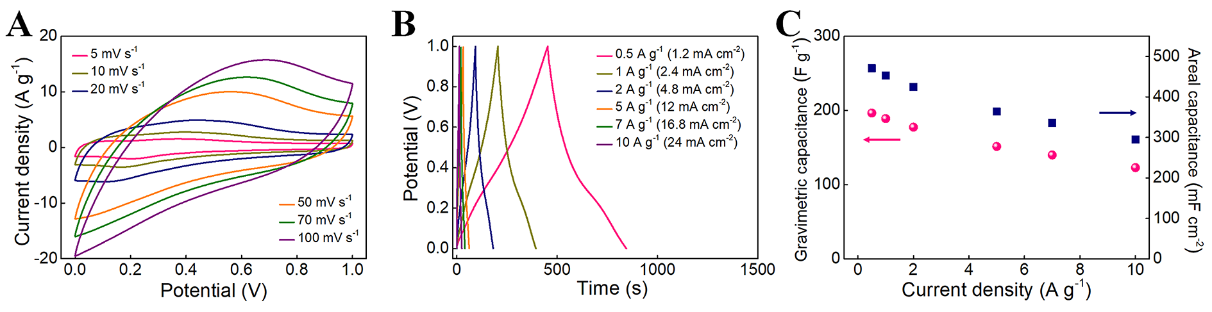


**Figure S21.** (A) CV curves of A-SC based on CPCH at various scan rates. (B) GCD curves of A-SC based on CPCH at various current densities. (C) Gravimetric and areal capacitances of A-SC based on CPCH at various current densities.

**Table S1.** Summary of [AA]/[EO] molar ratios of ICHs and PAA.

| Sample | Molar ratio of [AA]/[EO] |
| --- | --- |
| ICH-1 | 1.6/1 |
| ICH-2 | 1.3/1 |
| ICH-3 | 1/1 |
| ICH-4 | 1/1.3 |
| ICH-5 | 1/1.6 |
| PAA | 1/0 |

**Table S2.** Summary of ANI concentrations and pre-stretching strains for fabrication of SPCHs and CPCH.

| Sample | Concentration of ANI  [M] | Pre-stretching strain  [%] |
| --- | --- | --- |
| SPCH-1 | 0.18 | 300 |
| SPCH-2 | 0.36 | 300 |
| SPCH-3 | 0.54 | 300 |
| SPCH-4 | 0.72 | 300 |
| SPCH-a | 0.36 | 50 |
| SPCH-b | 0.36 | 100 |
| SPCH-c | 0.36 | 200 |
| SPCH-d | 0.36 | 400 |
| SPCH-e | 0.36 | 500 |
| CPCH | 0.36 | 0 |

**Table S3.** Summary of PANI contents in various SPCHs.

| Sample | Initial ANI concentration  [M] | Thickness of PANI layer  [μm] | Weight percentage of PANI  [wt%] | Areal loading of PANI  [mg cm^-2^] |
| --- | --- | --- | --- | --- |
| SPCH-1 | 0.18 | 126 | 1.4 | 1.25 |
| SPCH-2 | 0.36 | 153 | 2.6 | 2.40 |
| SPCH-3 | 0.54 | 169 | 3.4 | 3.05 |
| SPCH-4 | 0.72 | 176 | 3.6 | 3.30 |

**Table S4.** Summary of mechanical and electrochemical performances of SPCH-2 and conductive polymer-based composite hydrogels in literature.^[9-16]^

| Sample | | Tensile stress  [MPa] | Elongation at break  [%] | Strain [%]/Cycle | Specific capacitance [mF cm^-2^]/Cycle |
| --- | --- | --- | --- | --- | --- |
| APH-PANI | 0.3 | | 308 | - | 25.86/2000 |
| PANI-PVA/PHEA HGE | 1.07 | | 1467 | 100/2000 | 98/8000 |
| PANI/RGO-TA-GelMA-CNC | - | | - | - | - |
| PANI-PCH | 0.1 | | 290 | - | 488/10000 |
| PAA@Co^2+^-PANI | 0.017 | | 130 | - | 137.4/2000 |
| PVA/PA-PVA/PANI | 5.4 | | 714 | - | 85.3/- |
| PANI-based organogel | 0.27 | | 2250 | - | - |
| CNTs+PAA hydrogel | 0.16 | | 900 | - | 0.22/- |
| SPCH-2 | 0.26 | | 970 | 200/1500 |  |

**References**

[1] L. Lin, Y. Zhu, C. Li, L. Liu, D. Surendhiran, H. Cui, *Carbohydr. Polym.* **2018**, *198*, 225-232.

[2] V. R. Feig, H. Tran, M. Lee, Z. Bao, *Nat. Commun.* **2018**, *9*, 2740.

[3] J. Li, Z. Wang, L. Wen, J. Nie, S. Yang, J. Xu, S. Z. D. Cheng, *ACS Macro Lett.* **2016**, *5*, 814-818.

[4] N. Zhao, M. Li, H. Gong, H. Bai, *Sci. Adv.* **2020**, *6*, eabb4712.

[5] J. Han, G. Du, W. Gao, H. Bai, *Adv. Funct. Mater.* **2019**, *29*, 1900412.

[6] L. Li, Y. Zhang, H. Lu, Y. Wang, J. Xu, J. Zhu, C. Zhang, T. Liu, *Nat. Commun.* **2020**, *11*, 62.

[7] C. Wang, S. Yang, Q. Guo, L. Xu, Y. Xu, D. Qiu, *Chem. Comm.* **2020**, *56*, 13587-13590.

[8] J. Zou, S. Wu, J. Chen, X. Lei, Q. Li, H. Yu, S. Tang, D. Ye, *Adv. Mater.* **2019**, *31*, 1904762.

[9] W. Li, X. Li, X. Zhang, J. Wu, X. Tian, M.-J. Zeng, J. Qu, Z.-Z. Yu, *ACS Appl. Energy Mater.* **2020**, *3*, 9408-9416.

[10] J. Yang, X. Yu, X. Sun, Q. Kang, L. Zhu, G. Qin, A. Zhou, G. Sun, Q. Chen, *ACS Appl. Mater. Interfaces* **2020**, *12*, 9736-9745.

[11] H. H. Hsu, Y. Liu, Y. Wang, B. Li, G. Luo, M. Xing, W. Zhong, *ACS Sustain. Chem. Eng.* **2020**, *8*, 6935-6948.

[12] K. Wang, X. Zhang, C. Li, X. Sun, Q. Meng, Y. Ma, Z. Wei, *Adv. Mater.* **2015**, *27*, 7451-7457.

[13] H. Wang, L. Dai, D. Chai, Y. Ding, H. Zhang, J. Tang, *J. Colloid Interf. Sci.* **2020**, *561*, 629-637.

[14] Y. H. Wang, C. Lv, G. C. Ji, R. F. Hu, J. P. Zheng, *J. Mater. Chem. A* **2020**, *8*, 8255-8261.

[15] Y. Zhang, Y. Zhao, Z. Peng, B. Yao, Y. Alsaid, M. Hua, D. Wu, Y. Qiu, Q. Pei, X. Zhu, Z. He, X. He, *ACS Mater. Lett.* **2021**, *3*, 1477-1483.

[16] M. Hu, J. Wang, J. Liu, J. Zhang, X. Ma, Y. Huang, *Chem. Comm.* **2018**, *54*, 6200-6203.
